# Association between regular arrangement of collecting venules and *Helicobacter pylori* status in routine endoscopy

**DOI:** 10.1186/s12876-021-01960-w

**Published:** 2021-10-20

**Authors:** Cong Yuan, Xue-Mei Lin, Yan Ou, Lin Cai, Qian Cheng, Ping Zhou, Juan Liao

**Affiliations:** 1 Department of Gastroenterology, Affiliated Hospital of North Sichuan Medical College, Nanchong, 637000 Sichuan China; 2 Department of Pathology, Basic Medical College of North Sichuan Medical College, Nanchong, 637000 Sichuan China; 3 Department of Pathology, Affiliated Hospital of North Sichuan Medical College, Nanchong, 637000 Sichuan China; 4 Department of Gastroenterology, West China Forth Hospital, West China School of Public Health, Sichuan University, Chengdu, 610041 China; 5 Department of Pathology, West China Forth Hospital, West China School of Public Health, Sichuan University, Chengdu, 610041 China; 6 Non-communicable Diseases Research Center, West China-PUMC C.C. Chen Institute of Health, Sichuan University, Chengdu, 610041 China

**Keywords:** Helicobacter pylori, Gastrointestinal endoscopy, Gastric mucosa, Digestive system diagnostic techniques

## Abstract

**Background:**

The sensitivity of regular arrangement of collecting venules (RAC)-positive pattern for predicting *Helicobacter pylori* (*H. pylori*)-negative status greatly altered from 93.8 to 48.0% in recent two decades of various studies, while the reason behind it remained obscure. The aim of this study was to investigate the value of RAC as an endoscopic feature for judging *H. pylori* status in routine endoscopy and reviewed the underlying mechanism.

**Methods:**

A prospective study with high-definition non-magnifying endoscopy was performed. RAC-positive and RAC-negative patients were classified according to the collecting venules morphology of the lesser curvature in gastric corpus. Gastric biopsy specimens were obtained from the lesser and greater curvature of corpus with normal RAC-positive or abnormal RAC-negative mucosal patterns. *Helicobacter pylori* status was established by hematoxylin and eosin staining and immunohistochemistry.

**Results:**

41 RAC-positive and 124 RAC-negative patients were enrolled from June 2020 to September 2020. The prevalence of *H. pylori* infection in patients with RAC-positive pattern and RAC-negative pattern was 7.3% (3/41) and 71.0% (88/124), respectively. Among all 124 RAC-negative patients, 36 (29.0%) patients were *H. pylori*-negative status. Ten patients (32.3%) demonstrated RAC-positive pattern in 31 *H. pylori*-eradicated cases. The sensitivity, specificity, positive predictive value, and negative predictive value of RAC-positive pattern for predicting *H. pylori*-negative status were 51.4% (95% CI, 0.395–0.630), 96.7% (95% CI, 0.900–0.991), 92.7% (95% CI, 0.790–0.981), and 71.0% (95% CI, 0.620–0.786), respectively.

**Conclusions:**

RAC presence can accurately rule out *H. pylori* infection of gastric corpus, and *H. pylori*-positive status cannot be predicted only by RAC absence in routine endoscopy.

*Trial registration* The present study is a non-interventional trial.

## Background

*Helicobacter pylori* (*H. pylori*) infection has been involved in over 60% of people in the world [1], which is a well-known risk factor for gastric disorders including active gastritis, peptic ulcer, MALT lymphoma and adenocarcinoma [2]. Invasive and noninvasive diagnostic tests for *H. pylori* have been extensively used in the clinical practice [3–5]. Among these methods, endoscopic approach is a potential benefit, which can provide real-time mucosal findings and prompt targeted biopsy. In recent years, the technological developments in magnifying endoscopy have allowed accurate diagnosis of *H. pylori* infection by evaluating the pit and vascular patterns [4]. However, magnifying endoscopy is less commonly used in routine clinical practice, and the procedure requires more time and expenditure, and specialized training. Therefore, diagnosis of *H. pylori* by non-magnifying endoscopy would still be of great interest to the general endoscopists.

By using non-magnifying endoscopy, regular arrangement of collecting venules (RAC) in the gastric body is generally recognized as a characteristic feature of a normal stomach without *H. pylori* infection [6]. In 2002, Yagi et al. [6] study had indicated that the presence of RAC at the distal part of the lesser gastric curvature for predicting *H. pylori*-negative normal stomach had more than 90% sensitivity and specificity. RAC pattern becomes invisible when gastric body mucosa was affected by *H. pylori* infection [7]. However, in numerous subsequent studies between 2004 and 2019, RAC absence was associated with *H. pylori*-positive status in varied proportion from 47.3% to 94.4% among different cases [8–17]. Hence, the absence of RAC did not always indicate the positive status of *H. pylori*. So, the aim of our study was to investigate the prevalence of *H. pylori* in daily routine endoscopy without magnification, and evaluate the association between RAC pattern and *H. pylori* status.

## Methods

### Patients collection

We designed a prospective study including inpatients and outpatients who underwent upper GI endoscopy from June 2020 to September 2020. Consecutive patients with more than 18-years old were invited to attend the study. The following baseline characteristics were collected: age, sex, use of nonsteroidal anti-inflammatory drugs (NSAIDs), and history of *H. pylori* eradication in the last one year. Patients were excluded if they had previous partial gastrectomy, the diseases such as cirrhosis, chronic respiratory disorders, inflammatory bowel disease, collagen disease, and taken regular use of anticoagulants. The study protocol was approved by the institutional review board of West China Forth Hospital, Sichuan University (No. HXSY-EC-2020064). All participating patients gave written informed consent.

### RAC pattern classification of corpus by non-magnifying endoscopy

**Fig. 1:**
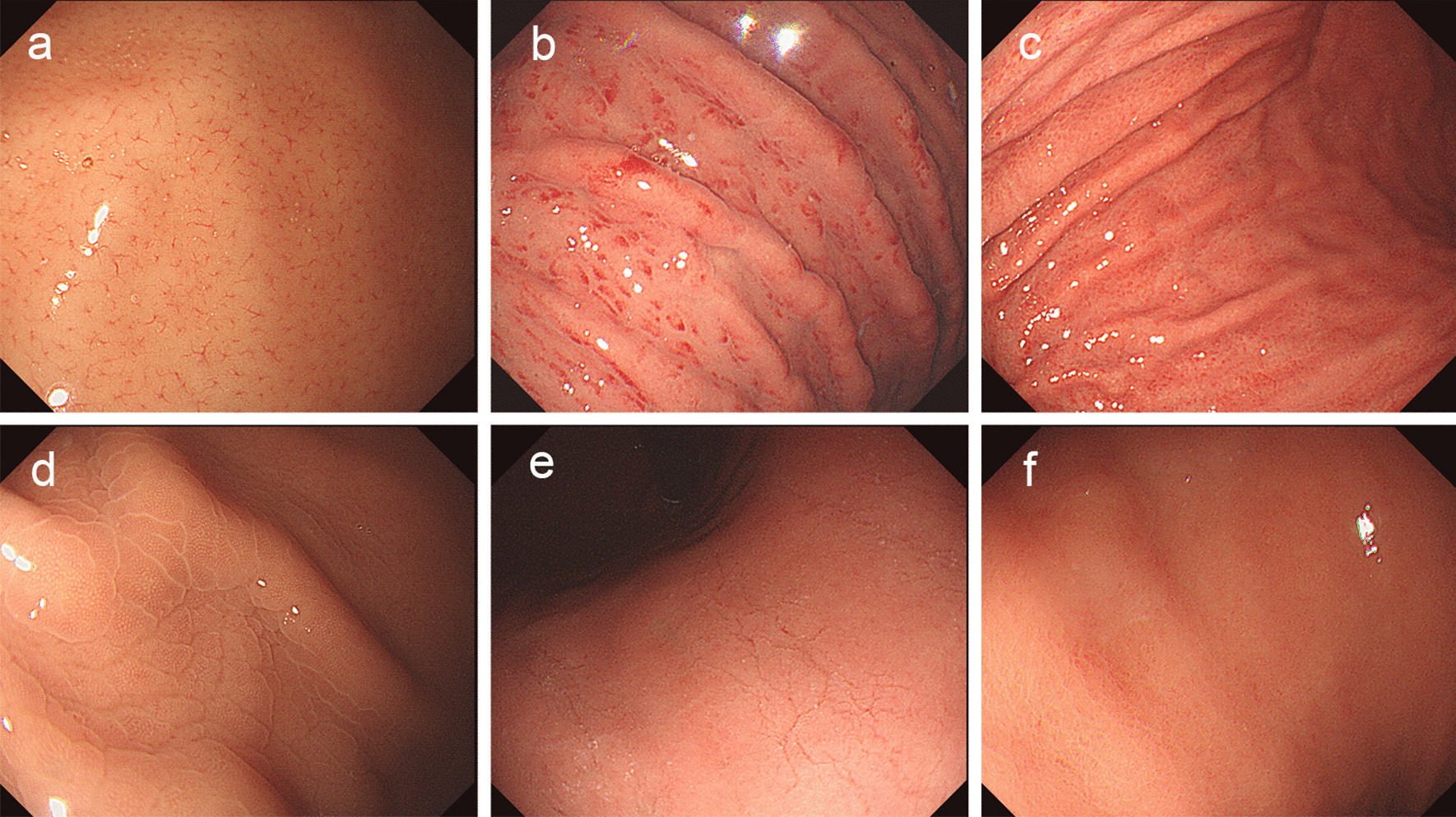
Normal RAC-positive pattern (**a**) and abnormal RAC-negative pattern (**b**–**f**) in the gastric corpus observed by non-magnifying endoscopy. **a** RAC-positive pattern showing numerous minute red dots with claw-like and regular intervals; **b** Spotty redness appearance; **c** Diffuse redness appearance; **d** Mosaic-like appearance; **e** Cleft-like appearance; **f** RAC-invisible mucosa showing untypical appearance with difficulties to classify

After routine examination of the whole stomach by high-resolution endoscopy (GIF-H290; Olympus Optical Co. Ltd., Tokyo, Japan), the close-up observation of corpus was performed with a distance no more than 10 mm between the endoscope tip and mucosal surface, as described previously [13]. The collecting venules (CVs) morphology at the lesser gastric curvature was classified [18]: regular arrangement of CVs as RAC-positive pattern (Fig. 1a), obscure and irregular arrangement of CVs as RAC-negative pattern. Some researchers further divided RAC-negative corpus mucosa into several types [3, 13–15, 19], such as spotty redness, diffuse redness, mosaic pattern, cleft-like appearance, untypical pattern, et al. (Fig. 1b-f).

### Biopsy specimens in the corpus and diagnosis of *H. pylori* infection

*H. pylori* infection in some patients only affected gastric antrum [6], and RAC appeared only in gastric body and fundus [7]. To ensure that *H. pylori* has infected the body of stomach, and explore the relationship between RAC pattern and corpus *H. pylori* status, two biopsy specimens were taken directly from the lesser curvature and greater curvature in the corpus, avoiding some certain areas such as erosion, ulceration and gastric mucosa with a suspicion of intestinal metaplasia. These two sites are recommended by the update Sydney System for gastric corpus biopsy [20], and the gastric body greater curvature is a better site to detect current *H. pylori* infections [21].

**Fig. 2:**
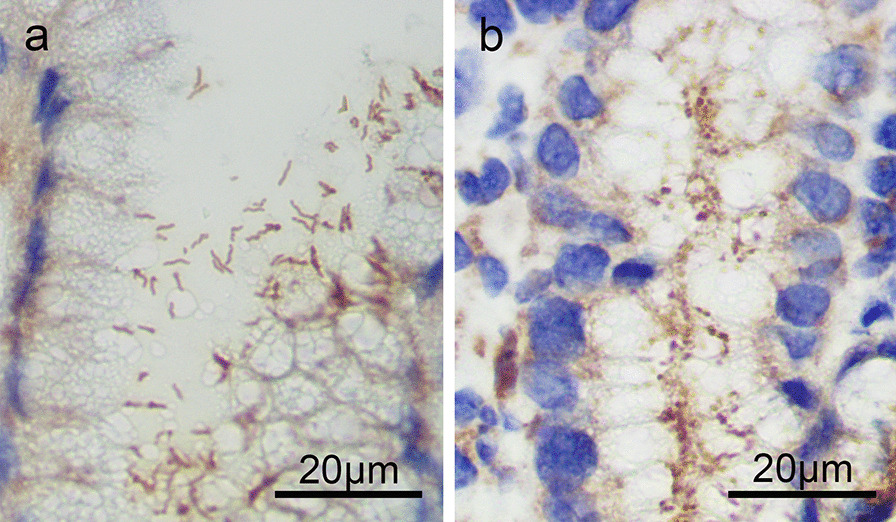
*H. pylori* detected by immunohistochemical stain (IHC). **a** The predominant bacilliform of *H. pylori* can be seen. **b** A large amount of coccoid *H. pylori* is visible. (IHC, × 1000 oil immersion lens)

The muscularis mucosae side of each biopsy specimen was stretched and fixed on filter paper, then bathed in a 10% formalin solution. 5-μm sections of paraffin wax embedded tissues were stained. A diagnosis of *H. pylori* infection was achieved if bacillary and/or coccoid *H. pylori* was identified on histopathological examination with H&E assay and/or monoclonal immunohistochemistry (IHC) (mouse monoclonal antibody, MX014, MXB Biotechnologies, Fuzhou, China). In general, only rod-shaped *H. pylori* can be identified in the H&E sections considering that coccoid *H. pylori* may mimic other bacteria or cell debris on H&E preparations [21]. To avoid the interference of impurities in IHC sections, more than 5 spherical *H. pylori* per high power field was identified as coccoid *H. pylori* positive (Fig. 2). All the assessment of gastric specimens was conducted by two pathologists, who were blinded to the clinical and endoscopic findings.

### Statistical analysis

With the assumption that the RAC would have a sensitivity of 90% with a confidence level of 95% and precision of 10%, a total of 158 patients were required. Continuous data with normal distribution were presented as mean ± SD, continuous data without normal distribution were presented as median and interquartile range (IQR) and for nominal variables data were presented as percentages. The normality was tested using the Kolmogorov–Smirnov test. Regarding the association between RAC pattern and *H. pylori* status, the sensitivity, specificity, positive predictive value (PPV), negative predictive value (NPV), and 95% confidence intervals (CI) were calculated [22]. Chi-square test and Mann–Whitney U-test were used to compare RAC-positive and RAC-negative group. The inter- and intraobserver reproducibility was calculated by using kappa values as described by Landis and Koch [23]. A *P* value of less than 0.05 was required for significance. All statistical analyses were performed using SPSS statistical software (version 22 SPSS Inc., Chicago, US).

## Results

### Study subjects and baseline characteristics

Demographics and endoscopic mucosal patterns were summarized in Table 1. A total of 165 patients were enrolled with a median age of 52 years (IQR 41, 61; range 18–79 years), and 107 (64.8%) patients were male. The age distribution of RAC-positive and RAC-negative groups was different, and RAC was more common in cases under 50 years old (*P* < 0.05). There was no difference in RAC between NSAIDs user and non-user groups (*P* < 0.05). 10 patients (32.3%) demonstrated RAC-positive pattern in 31 *H. pylori*-eradicated cases.

**Table 1:** Baseline characteristics of the patients in current study

Characteristics	Total (n = 165)	RAC positive (n = 41)	RAC negative (n = 124)	*P* value
Age, years (median, IQR)	52 (41, 61)	40 (30, 51)	49 (37, 58)	0.005
Age				
< 50 years	89 (53.9)	29	60	0.01
≥ 50 years	76 (46.1)	12	64	
Sex				
Male	107 (64.8)	28	79	0.59
Female	58 (35.2)	13	45	
NSAIDs				
Yes	31 (18.8)	11	20	0.39
No	100 (60.6)	36	64	
Unknown	34 (20.6)			
*H. pylori* status				
Positive	91 (55.2)	3	88	< 0.001
Negative	74 (44.8)	38	36	
*H. pylori* eradicated				
Yes	31 (18.8)	10	21	0.48
No	101 (61.2)	26	75	
Unknown	33 (20.0)			

*IQR* interquartile range, *NSAIDs* nonsteroidal anti-inflammatory drugs, *RAC* regular arrangement of collecting venules

### *H. pylori* infection status in RAC-positive and RAC-negative pattern

*H. pylori* positive rate was different between RAC-positive and RAC-negative group (7.3% versus 71.0%, *P* < 0.05). In RAC-positive group, *H. pylori*-negative status was revealed in 92.7% (38/41) patients. In RAC-negative group, 29.0% (36/124) of patients were *H. pylori*-negative status. Among 88 (71.0%, 88/124) *H. pylori*-positive patients in RAC-negative group, 80 had both spherical and rod shape, 5 had only spherical shape and 3 had only rod shape. In RAC-negative subgroups, the *H. pylori* positive rate was 80.6%, 80.0%, 81.6%, 52.6% and 37.5% for spotty redness, diffuse redness, mosaic pattern, cleft-like appearance, and an untypical pattern, respectively (Table 2). RAC presence at the lesser gastric curvature was associated with a 51.4% sensitivity (95% CI, 0.395–0.630) and a 96.7% specificity (95% CI, 0.900–0.991) for estimating *H. pylori*-negative status. PPV and NPV were 92.7% (95% CI, 0.790–0.981) and 71.0% (95% CI, 0.620–0.786), respectively.

**Table 2:** *H. pylori* infection status in endoscopic mucosal patterns

Mucosal patterns	Total	*H. pylori* status (n, %)		
		Negative	Positive	*P* value
RAC positive	41	38 (92.7)	3 (7.3)	< 0.001*
RAC negative	124	36 (29.0)	88 (71.0)	
Spotty redness	31	6 (19.4)	25 (80.6)	
Diffuse redness	20	4 (20.0)	16 (80.0)	
Mosaic	38	7 (18.4)	31 (81.6)	
Cleft	19	9 (47.4)	10 (52.6)	
Untypical pattern	16	10 (62.5)	6 (37.5)	

*RAC* regular arrangement of collecting venules. *P* < 0.001* RAC-positive versus RAC-negative group

### Inter- and intraobserver agreement assessment

The k-values for inter- and intraobserver agreement for the endoscopic mucosal patterns were significant. The k-values for inter- and intraobserver agreement for the assessment of *H. pylori* status were also significant (Table 3).

**Table 3:** Inter- and intraobserver agreement

	Interobserver agreement			
	% agreement	k value (95% CI)	% agreement	k value (95% CI)
RAC pattern	85.6	0.74 (0.71–0.78)	89.4	0.88 (0.78–0.96)
*H. pylori* status	92.7	0.86 (0.80–0.92)	93.5	0.94 (0.87–0.98)

The k-values for inter- and intraobserver agreement for mucosal patterns and *H. *pylori status were significant

*CI* confidence interval

## Discussion

RAC presence has been well known as a characteristic endoscopic feature in *H. pylori*-negative normal stomach [6, 7]. In 2002, Yagi et al. [6] researchers have indicated that the presence of RAC for predicting *H. pylori*-negative normal stomach had 93.8% sensitivity and 96.2% specificity. In many subsequent studies (Table 4) [8–17], the RAC-positive pattern also demonstrated a high specificity between 85.7% and 100%, however, the sensitivity of RAC-positive pattern varied from 93.5% to 48.0%. In our study, RAC presence has only 51.4% sensitivity and 71.0% NPV for predicting *H. pylori*-negative status in routine clinical practice despite a good specificity (96.7%) and PPV (92.7%). These studies strongly supported the idea that the presence of RAC in the lesser corpus can accurately identify *H. pylori*-negative gastric body mucosa [24], but RAC absence did not always point out *H. pylori*-positive corpus mucosa.

**Table 4:** Summary of study characteristics on the association of RAC and *H. pylori* status

Author	Year	Reference methods								Number of patients	Eradicated patients	Sensitivity (%)	Specificity (%)
		Histology		RUT		UBT	Serology	Culture					
		Corpus	Antrum	Corpus	Antrum			Corpus	Antrum				
Yagi et al. [6]	2002	√	√	√	√			√	√	557	Unknown	93.8	96.2
Nakayama et al. [8]	2004	√	√	√	√	√	√			52	Excluded	93.5	100
Anagnostopoulos et al. [9]	2007	√	√	√	√					95	Unknown	92.8	100
Machado et al. [10]	2008		√		√					99	Excluded in 12 months	88.1	96.9
Gonen et al. [11]	2009	√	√	√	√					129	Excluded	82.8	85.7
Yan et al. [15]	2010	√		√						112	Excluded	66.7	100
Hidaka et al. [12]	2010	√	√			√	√			87	Excluded	86.7	100
Cho et al. [13]	2013	√	√	√	√					617	Excluded	89.1	93.3
Kato et al. [14]	2013	√								275	Excluded	48.0	93.6
Tongtawee et al. [16]	2015	√		√						200	Excluded in 2 months	53.0	100
Garces-Duran et al. [17]	2019	√	√	√	√					140	Included	49.0	100
Yuan et al.*	2020	√								165	Included	51.4	96.7

Corpus and antrum denoting the corresponding biopsy site

*UBT* urea breath test, *RUT* rapid urease test

*The results in current study

In routine endoscopy, many conditions can cause the disappearance of RAC, including *H. pylori*-related factors (such as chronic active gastritis caused by current *H. pylori* infection, and chronic inactive gastritis after *H. pylori* eradication), and *H. pylori*-unrelated factors (such as *H. pylori*-negative gastritis, and gastropathy induced by liver cirrhosis, et al.) [6, 25–28]. Shiota et al. [29] and Nordenstedt et al. [30] had found *H. pylori*-negative gastritis in 17.7% and 21% of patients with histologic gastritis. Although *H. pylori*-negative gastritis was a condition that cannot be ignored, the diagnosis process was complicated and difficult to apply in daily work [29, 30]. Thus, we have not further evaluated such patients with *H. pylori*-negative gastritis in the present study. In inclusion criteria, the patients with liver cirrhosis have been excluded. In addition, NSAIDs were also one of the common causes of gastropathy. Our results revealed that no significant differences in RAC pattern were found in a small group of patients treated with NSAIDs (Table 1), which was consistent with previous studies [17].

In view of the high prevalence of *H. Pylori*, *H. pylori*-related factors are the lead cause of RAC disappearance. RAC absence can be happened in the patients with current or past *H. Pylori* infection [26, 31]. In suspicious patients with chronic active gastritis, in addition to the disappearance of RAC, the accuracy of *H. pylori* status can be further improved by combining with other endoscopic manifestations, such as diffuse redness, spotty redness and swelling of areae gastricae [14]. *H. pylori*-eradicated cases have been arising considering the preventive effect of *H. pylori* eradication therapy for gastric cancer [32]. However, RAC will not reappear immediately after *H. pylori* eradication. Yagi et al. [26] have revealed that the RAC did not recover for over one year in 68% subjects after successful *H. pylori* eradication. Garces-Duran et al. [17] also found that about half of past *H. pylori*-eradicated patients were RAC-negative. Thus, RAC would be invisible in a considerable proportion of *H. pylori*-eradicated patients. We found that RAC was still absent in about two-thirds of patients (67.7%, 21/31) with *H. pylori* eradication history. These results revealed that RAC absence did not always indicate *H. pylori*-positive status in patients with *H. pylori* eradication history. Although detailed medical records were very vital for identifying the eradicated cases, some patients still failed to provide past *H. pylori* eradication clearly. In addition, partially eradicated patients may come from unintended *H. pylori* sterilization because of the infectious diseases in other organs. Besides, *H. pylori* may be also naturally eradicated without bactericidal therapy. These conditions made it difficult for us to accurately judge the past infection of *H. pylori* in clinical practice. Therefore, we investigated the total *H. pylori* prevalence in RAC-negative patients, and found that only 71.0% (88/124) subjects were *H. pylori-*positive. 29.0% (36/124) of patients were *H. pylori*-negative status despite the fact that RAC disappeared, which resulting in the low sensitivity (51.4%) and NPV (71.0%).

In these reports (Table 4) [6, 8–17] on the relationship between RAC pattern and *H. pylori* status, the sensitivity of RAC varied greatly from 93.8% to 48.0%, which may be related to different baseline characteristics of the patients and multiple methods of *H. pylori* status judgment. Among the 11 studies, four studies [9, 11, 13, 17] simultaneously detected *H. Pylori* in gastric body and antrum by histological examination and rapid urease test (RUT), and showed that the sensitivity of RAC decreased from 92.8 to 49.0% due to the difference in baseline characteristics. For example, in Garces-Duran et al. [17] study the patients with NSAIDs usage and past *H. pylori* eradication were enrolled, and the sensitivity was only 49.0%. In the present study, we did not also exclude analogous patients and the sensitivity was low (51.4%). Even if the patients with *H. pylori* eradication history were excluded in inclusion criteria, the sensitivity was only 66.7% in Yan et al. [15] study and 48.0% in Kato et al. [14] study. Indeed, in clinical work, it is very difficult for us to completely exclude insidious past *H. pylori* infection, especially unintended H. pylori sterilization and spontaneous eradicators mentioned above. Interestingly, the two studies [14, 15] only detected *H. pylori* in gastric body by histology and/or RUT. Therefore, the prevalence of *H. pylori* could only reflect the *H. pylori* status in the corpus, which was similar to our study. One studies [10] merely detected *H. pylori* in the antrum by histology and RUT, which can not reveal the infection of *H. pylori* in gastric body because in some patients the infection only affected gastric antrum [6]. Indeed, it was not appropriate to use the RAC in the body to predict *H. pylori* status in the antrum in this study.

The corrosion casting and scanning electron microscopy of blood vessels in gastric corpus mucosa revealed that CVs gradually formed at the level of gastric foveolar layer and descend to join the submucosal plexus, without receiving any further capillary tributaries on their course (glandular layer) [33–35]. The CV and the drainage vein below showed a tree-like stereostructure [36]. The morphologic changes or destruction of gastric foveola and gastric body glands can affect the arrangement and shape of CVs. In normal gastric body mucosa, the length of gastric foveola is about 200 μm or less [37, 38], which is within the penetration depth of the endoscopic illumination light [39]. Therefore, regular arrangement of CVs (RAC) can be seen in normal gastric body mucosa by gastroendoscopy.

RAC absence is one of the main manifestations of chronic active gastritis due to *H. pylori* current infection [6, 7, 12]. The gastric mucosal active inflammation should relieve shortly after eradication of *H. pylori*, however, RAC was still negative in some patients [17, 26]. Mild chronic inflammation in gastric mucosa can persist for more than 5 years after successful *H. pylori* eradication therapy in up to one-fifth of patients [40]. The appearance of RAC was the endoscopic manifestation of normal gastric corpus mucosa without pathologic changes [41, 42]. Saghier et al. [43] study has showed that foveolar length of corpus mucosa in *H. pylori* gastritis patients was significantly increased than that of normal *H. pylori*-uninfected gastric corpus. In a recent study from our team [38], we found that the prolongation of gastric foveolae could result in the invisibility of RAC. We revealed that in addition to *H. pylori* current infection, chronic inactive gastritis in *H. pylori*-eradicated patients can also cause RAC disappearing through the prolongation of gastric foveolae. Therefore, along with the increase of *H. pylori*-eradicated patients, the subjects with RAC-negative and *H. pylori*-negative entity are further accumulating, which can lead to decreased sensitivity. Hence, only RAC disappearance was no longer a reliable feature to judge *H. pylori*-positive status in daily practice. In fact, in Yagi et al. [6] study, the predicting corpus mucosa of RAC presence was not only *H. pylori*-negative status but also normal pathological features. However, in numerous studies [8–17], only *H. pylori* status was evaluated, ignoring gastric mucosa pathological abnormality, in particular the changes of gastric foveolae length. In a word, RAC presence was the endoscopic manifestation of normal gastric corpus mucosa with normal histology [41, 42], which can not only exclude *H. Pylori* infection, but also eliminate the pathological abnormalities of corpus mucosa caused by other factors.

Our study had several limitations: Firstly, our study only explored the relationship between RAC and *H. Pylori* infection in gastric corpus mucosa, and *H. pylori* status of gastric antrum mucosa was not evaluated. Therefore, *H. Pylori* status of gastric corpus cannot be represented the entire stomach. Secondly, histological detection of *H. pylori* can reduce the accuracy because of patchy distribution of the bacteria. Combination of multiple methods was helpful to more accurate detection of *H. Pylori* such as urea breath test, serological examination, PCR and culturing. However, PCR and *H. pylori* culturing was not convenient in daily clinical practice. In addition, urea breath test and serological examination cannot distinguish the patients in which the gastric body was only affected, and not be performed in the present study. Thirdly, RAC accuracy may be affected by patient age [44]. We did not conduct age stratification analysis due to the limited sample size. Lastly, this was a single-center study, with a limited number of cases and a short time span. In future, more patients can be included to analyze different detective methods of *H. pylori* and different patient subgroups to strengthen our results.

## Conclusions

The present study has demonstrated that RAC presence can accurately rule out the *H. pylori* infection of gastric corpus, but the positive status of *H. pylori* cannot be effectively predicted only by the absence of RAC in routine endoscopy. In patients with RAC-negative pattern, other endoscopic features such as diffuse redness, spotty redness and swelling of areae gastricae et al. should be combined to improve the diagnosis of *H. pylori* status.

## Data Availability

All data analyzed during this study are included in this published article.

## Abbreviations

*H. pylori*: *Helicobacter pylori*
RAC: Regular arrangement of collecting venules
CVs: Collecting venules
IHC: Immunohistochemistry
IQR: Interquartile range
NSAIDs: Nonsteroidal anti-inflammatory drugs
CI: Confidence interval
PPV: Positive predictive value
NPV: Negative predictive value
RUT: Rapid urease test
UBT: Urea breath test
